# Cost-effectiveness of US National Institute of Health and European Union FP7 Projects on Active Ageing and Elderly Quality of Life-Author's reply

**DOI:** 10.2174/1745017901915010010

**Published:** 2019-01-23

**Authors:** Mauro Giovanni Carta, Michela Atzeni, Alessandra Perra, Quirico Mela, Martina Piras, Giorgia Testa, Germano Orrù, Iskren Kirilov

**Affiliations:** 1 Department of Medical Sciences and Public Health, University of Cagliari, Cagliari, Italy; 2 Innovation Sciences and Technologies, University of Cagliari, Cagliari, Italy; 3 Department of Surgical Sciences, University of Cagliari, Cagliari, Italy

## Abstract

**Background:**

The use of bibliometric analysis to assess scientific productivity and impact is particularly relevant for EU funding programs. The objective of the present study is to assess the impact on scientific literature by focusing specifically on the cost-effectiveness of FP7 and NHI projects in the fields of AA and QoL, respectively.

**Methods:**

Twenty projects were randomly selected from the CORDIS database in accordance with the following criteria: funded by the FP7; accepted from 1^st^ January 2007 to 31^st^ December 2012; concluded by 31^st^ August 2017;

For each project selected, we determined: number of publications in Scopus and Google databases attributable to the project; number of papers published in Q1 quartile of the SCIMAGO rank; number of citations found in Scopus and Scholar Google; amount of funds allocated.

**Results:**

The study has confirmed the results of the previous one, namely that the number of publications and the number of citations per project on active ageing are similar in projects funded by the NHI in the United States and those funded by the FP7 in Europe. However, when it comes to cost-effectiveness, it results that European projects have a cost ten times higher than the Americans ones.

**Conclusion:**

Our study shows lower cost-effectiveness of FP7-European projects than the American-NIH on active aging. The results of this research, albeit with the limits already outlined, will have to be taken into consideration in the evaluative research of the future.

## 1 INTRODUCTION

A study recently evaluated the impact on scientific literature of a sample of projects funded by the 7th European Union’s Framework Program for Research and Innovation (FP7) on Active Ageing (AA) and elderly quality of life (QoL) in comparison to the same number of projects funded by the USA’s National Institute of Health (NHI) [1]. The results show that the European and American projects have a similar impact in terms of a number of published articles and number of citations by published papers per single project.

This study was part of a broader one aiming to set up a methodology that allows measuring the impact of European projects funded by Horizon 2020 [2] and the subsequent EU framework programs for research and innovation. The use of bibliometric analysis to assess scientific productivity and impact is particularly relevant for the EU funding programs, in particular, in relation to the promotion of so-called open science (especially, open access to publications and scientific data) and also in relation to the importance they have in the market created innovation.

The choice of the scientific domain of AA and QoL is motivated by the fact that it has been one of the priority biomedical areas of FP7 supported with a total budget of €115 million [3].

The number of Europeans aged over 65 is expected to nearly double, from 85 million in 2008 to 151 million by 2060, and the number of those over 80 is expected to rise from 22 to 61 million in the same period. Research furthering lifelong health, active ageing and well-being will, therefore, be a cornerstone in the successful adaptation of societies to this demographic change [4].

A recent commentary on the previous study acknowledged interest in the results presented while highlighting the need to introduce costs of the evaluated projects as an independent variable in future researches [5]. The commentary suggests “The Horizon / FP7 calls to finance projects on average larger than NIH. If there were any disparities in the funds collected, this would not show an equal impact between the two shores of the Atlantic. This hypothesis must be verified” [5].

The commentary also introduced some elements that would suggest a greater impact on US publications. The authors state that the US is the world leader in terms of publications. They also recall that none of the 20 top scientific journals in Scimago ranking is European, and also the fact that, among the first 100 scientific journals of the SCIMAGO ranking, only 12 are European [6]. Moreover, recently published evidence suggests that in some countries of the European Union, the performance of scientific researchers does not have the same value for academic careers [7, 8]. In the field of psychosocial interventions, an analysis of some systematic reviews indicates a greater impact of American literature compared to that of Europe [9-12].

The objective of the present study is to assess the impact on scientific literature and cost-effectiveness of FP7 and NHI projects in the fields of AA and QoL, respectively.

## 2 METHODS

### 2.1 Design

Observational design.

### 2.2 Study Sample

Twenty projects were randomly selected from the CORDIS database in accordance with the following criteria:

1. Funded by the FP7;
2. Accepted from 1^th^ January 2007 to 31^th^ December 2012.
3. Concluded by 31^th^ August 2017.

As keywords for the search, we used “active ageing” and “ageing and quality of life”.

With the same criteria and keywords for the search, twenty projects funded by the US NHI were selected. A block was built for each EU project which included all eligible NHI projects funded ± 1-year with the matched EU project. Then 20 US projects were randomly selected, one from each block. The selected projects were automatically excluded when extracted.

### 2.3 Variables, Search for Publications and Measure of Citations

For each selected project, we determined:

1. The number of publications found in the Scopus and Google databases attributable to the project, by introducing the project title, the keywords of the project, the name of the principal investigator into the two databases (Scopus and Scholar), and then verifying the correct link between each found paper and the project;
2. Using the same method and criteria, we calculated the number of papers published in journals classified in the Q1 quartile of the SCIMAGO rank for each of the two groups;
3. For each publication and for each project, we calculated the number of citations found in Scopus and Scholar Google databases;
4. For each project, we found the amount of funds allocated.

The search of articles and relative citations referred to August 2017 (for producing data comparable with the previous research).

The search of papers was carried out using the name of the principal investigator, the names of the official collaborators of the project and the title of the project. Furthermore, all principal investigators were contacted by e-mail requesting information about any ongoing publications or other publications in a journal indexed in Scopus or Scholar that we may not have found.

The search for publications and citations was carried out in the blind by two independent researchers as well as verification of the correspondence of each publication to a project. A third researcher was invited to decide, in case of disagreement.

### 2.4 Data Analysis

The comparison will be processed using the one-way Analysis Of Variance (ANOVA) as the variables were measured on numeric scales. Nominal data were analyzed by means of the chi-square test.

### 2.5 Ethics

The study was conducted according to the principles of the Helsinki Declaration. The ethics committee of the Azienda Mista Ospedaliero Universitaria di Cagliari approved the study. All results of this study will be granted open access.

## 3 RESULTS

Table **1** shows the results concerning all indicators in the two samples (as the mean and standard deviation in the overall sample); Table **2** shows the results of comparisons. The two groups were homogeneous in all the indicators considered in the previous research (mean number per project of articles published in journals indexed in Scopus; mean number of articles per project published in journals ranked in the first quartile of quality (Q1) in Scimago; mean number of citations per project in journals indexed in the Scopus; mean number of citations per project in journals indexed in Google) with any difference between European and US projects.

The projects for which the publications showed no citations in the journals indexed in the Scopus depository were 7 (35.0%) in the European sample and 2 (10.0%) in the American sample; this difference reaches the limit of statistical significance (χ2 = 0.59; p=0.05). The projects for which their publications did not show any citations in the journals indexed in the Google depository were 5 (20.0%) in the European sample and 2 (10.0%) in the American sample; this difference did not reach statistical significance (χ2 = 0.15; p=0.21).

The new indicator introduced the average budget per project showed a marked difference between the two groups. The mean budget of each project funded by the UE was 3,785,513 dollars against 315,813 dollars of each project funded by the NIH.

If we consider the total of 104 publications of the NIH project, the total of 114 publications of EU/7WP EU and the total cost of the 20 projects (NIH = $3,472,149; EU/7WP EU = Euro 44,543,630), the cost per publication is 33,380 dollars in the US and 390,733 euros in the UE).

## 4 DISCUSSION

The study confirms the results of the previous one, namely the fact that the number of publications and the number of citations per project on active ageing are similar between projects funded by the NHI in the United States and those funded by the FP7 in Europe. However, when it comes to cost-effectiveness, it results that European projects have a cost ten times higher than the Americans ones.

Owing to the size of the sample, these results should be taken with caution and considered mainly as a stimulus to go further into this research, including other indicators, and also extending it to other priority scientific areas.

Interpretation of results must be done in the light of the specificities of each program. Thus, the American programs appear to be more basic research-oriented, while the European ones are more focused on applied research and turning scientific results into innovative marketable products and services [13, 14]. On the other hand, without the presence of solid results, the European Medicines Agency (EMA) in Europe or the Food and Drug Administration (FDA) in the US [15] cannot approve innovative treatments or technologies. Therefore, the objective of supporting market competitiveness is strictly linked, if it does not coincide, with the aim of publishing a lot and with a strong impact on literature (ie attracting many citations). But in addition to needing a minimum of published evidence to file for patents or market, the more treatment is supported by scientifically robust published evidence, the more competitive it will be.

However, the modus operandi and rules of participation in both programmes are different. In the case of EU programs, the standard implementation mode is collaborative research that requires participants from at least three different countries, among EU Member states and associated countries. Moreover, the EU programs are not intended to replace national ones, but rather to support the achievement of political objectives, mainly completion of the European research area inspired by the European single market, in which researchers, scientific knowledge and technology circulate freely (as stated in Title XIX of the Treaty on the Functioning of the European Union).

In terms of expenditure on research and innovation (R&I), Europe still lags behind its main competitors. Thus, in 2015, in accordance with the OECD, Europe spent 1.96% of its GDP against 2.07% in China and 2.69% in the US. In the case of China, there has been a 60% growth in R&I expenditure from 2000 to 2015, while in Europe, the growth was only 15% [16, 17]. It would be worth mentioning that achieving 3% of the EU's GDP invested in R&I is one of the targets of the Europe 2020.

Regarding scientific publications, the European Union, considered as the sum of its member states, maintains its excellence of production, both in terms of quality and quantity. Nevertheless, the European countries have been losing positions, in particular in some emerging scientific areas, including the biomedical. In 2017, only Germany, Italy and Poland maintained their SCIMAGO ranking for scientific production which they achieved 10 years ago (fourth, eighth and eighteenth), while others, such as France, Spain, the Netherland, Sweden, Belgium and Austria have stepped down in this ranking [1]. In addition, the number of research centers in the world in the top 100 dropped from 14 in Europe to only 10 in 2018 [1].

## CONCLUSION

Our study shows lower cost-effectiveness of the FP7 European projects compared to the American NIH on active aging. This poorer outcome was found only in relation to cost of the researchers at the equal impact on scientific literature.

The differences between the EU and the US research and innovation landscape and policies may be a determinant on the difference in the costs of projects.

However, the results of this research, albeit with the limits already outlined, will have to be taken into consideration in the evaluative research of the future.

## Figures and Tables

**Table 1A:** Projects of US NIH.

Projects found	Budget$$	N° of Papers	Paper sin Q1	Citations in Scopus	Citations in Scholar
**I**	67586	2	1	0	79
**II**	120044	13	10	22	349
**III**	375665	9	8	18	358
**IV**	790162	14	8	81	196
**V**	365388	14	13	347	684
**VI**	576731	13	10	480	675
**VII**	213319	3	3	8	103
**VIII**	386231	4	1	14	101
**IX**	83151	4	2	72	97
**X**	548595	0	0	0	0
**XI**	3337	1	1	21	42
**XII**	4131	13	10	165	309
**XIII**	292708	4	3	56	93
**XIV**	255456	0	0	0	0
**XV**	743102	12	10	92	117
**XVI**	540003	7	4	908	1668
**XVII**	531687	1	1	27	51
**XVIII**	89974	4	3	72	130
**XIX**	62730	16	8	143	246
**XX**	266264	6	3	23	40

**Table 1B:** Projects of EU.

Budget	N° of Papers	Papers in Q1	Citations in Scopus	Citations in Scholar
€5.975.821,00	6	3	198	390
€3.320.457,00	2	1	0	0
€5.980.000	7	1	34	57
€2.820.000	42	25	689	1108
€2.450.000	16	6	34	77
€3.423.572	0	0	0	0
€399.360	0	0	0	0
€5.999.548	1	1	380	555
€5.781.957	11	9	185	283
€587.150	0	0	0	0
€800.000	10	1	43	92
€2.749.338	4	1	13	22
€2.250.000	3	2	95	158
€950.000	0	0	0	0
€1.056.427	12	3	116	219
€ 5.906.757	19	13	424	668
€ 3.484.788	0	0	0	0
€ 2.350.758	12	12	242	419
€ 2.989.877	7	3	6	27
€ 4.406.272	1	1	0	2

**Table 2:** Comparisons Between EU And US Projects.

–	N° of Papers	Papers in Q1	Citations in Scopus	Citations in Scholar	Budget (Dollars)
US Projects	7.00±5.44	4.95±4.17	127.45±221.16	266.9±384.36	315,813.2±242,656.54
EU Projects	7.65±9.95	4.10±4.30	122.95±175.82	203.85±294,72	3,785,513±2308876.84
F(1, 38,39)	0.066	0.403	0.005	0.339	**44.673**
P	0.799	0.529	0.944	0.564	**<0.0001**

## References

[1] Kirilov I., Atzeni M., Perra A., Moro D., Carta M.G. (2018). Active Aging and Elderly’s Quality of Life: Comparing the Impact on Literature of Projects Funded by the European Union and USA.. Clin. Pract. Epidemiol. Ment. Health.

[2] European Commission https://ec.europa.eu/programmes/ horizon2020/.

[3] European Commission https:// ec.europa.eu/ research/ fp7/ index_en.cfm ? pg = sitemap # themes.

[4] Bramanti A., D’Aloja E., Cabras F., Paribello P., Moro M.F., Lindert J., Carta M.G. (2018). The Elderly and the City: Lack of Knowledge on Violence Perception and Consequences on Daily Life.. Clin. Pract. Epidemiol. Ment. Health.

[5] Sancassiani Federica, Petretto Donatella Rita, Romano Ferdinando, Preti Antonio (2018). Exploring physical and psychosocial well-being and self-awareness as a new frontier in active aging.. Clinical Practice & Epidemiology in Mental Health.

[6] SCIMAGO. https://www.scimagojr.com.

[7] Carta M.G. (2015). Why has scientific productivity increased in Italy?. Lancet.

[8] Preti A. (2017). Italian Abilitazione Scientifica Nazionale.. Lancet.

[9] Mura G., Carta M.G. (2013). Physical activity in depressed elderly. A systematic review.. Clin. Pract. Epidemiol. Ment. Health.

[10] Mura G., Sancassiani F., Machado S., Carta M.G. (2014). Efficacy of exercise on depression: A systematic review.. Int. J. Psychosoc. Rehabil..

[11] Sancassiani F, Pintus E, Holte A, Paulus P, Moro MF, Cossu G, Angermeyer MC, Carta MG, Lindert J (2015). Enhancing the Emotional and Social Skills of the Youth to Promote their Wellbeing and Positive Development: A Systematic Review of Universal School-based Randomized Controlled Trials.. Clin Pract Epidemiol Ment Health..

[12] Agabio R, Trincas G, Floris F, Mura G, Sancassiani F, Angermeyer MC (2015). A Systematic Review of School-Based Alcohol and other Drug Prevention Programs.. Clin Pract Epidemiol Ment Health..

[13] European Commission - Commission Staff Working Document (2016). Horizon 2020 Annual Monitoring Report 2015..

[14] Matthews H. (2005). The 7^th^ EU research framework programme.. Nanotechnol. Percept..

[15] (2018). https://www.ema.europa.eu/documents/report/annex-1-detailed-information-conditional-marketing-authorisations_en.pdf.

[16] Kneipp S.M., Schwartz T.A., Drevdahl D.J., Canales M.K., Santacroce S., Santos H.P., Anderson R. (2018). Trends in Health Disparities, Health Inequity, and Social Determinants of Health Research: A 17-Year Analysis of NINR, NCI, NHLBI, and NIMHD Funding.. Nurs. Res..

[17] http://www.oecd.org/innovation/inno/researchanddevelopmentstatisticsrds.htm.

